# Supplementary material for: Feasibility and acceptability of collecting passive phone usage and sensor data via Apple SensorKit
Source: PLoS One. 2025 Aug 13;20(8):e0329707. doi: 10.1371/journal.pone.0329707 (PMC12349082; doi:10.1371/journal.pone.0329707)
Supplement: S1 Table — aResidents were coded as underrepresented in medicine according to the American Association of Medical Colleges definition as “racial and ethnic populations that are underrepresented in the medical profession relative to their numbers in the general population.” In this study, this group included interns self-identifying as African American, Arab or Middle Eastern, Latino, Native American, Pacific Islander, other, or multi-racial. bSurgical specialties were assigned based on the American College of Surgeons classification. Specifically, for this study, physicians in the following specialties were classified as “surgical”: General Surgery, Gynecology and Obstetrics, Neurological Surgery, Orthopaedic Surgery, Otolaryngology, Plastic Surgery, Urology, and Other surgical. Physicians from the following specialties were classified as “non-surgical”: Internal Medicine, Pediatrics, Psychiatry, Neurology, Emergency Medicine, Internal Medicine-Pediatrics, Family Medicine, Family Practice, Anesthesiology, Dermatology, Medical Genetics, Nuclear Medicine, Pathology, Physical Medicine & Rehabilitation, Preventative Medicine, Radiation Oncology, Radiology-Diagnostic, Sleep Medicine, and Other Non-surgical. (DOCX) [file pone.0329707.s001.docx]

**Supporting Information**

Supplementary Table 1. Demographic Characteristics (n=695)

| Gender | n (%) |
| --- | --- |
| Women | 396 (57.0) |
| Men | 298 (42.9) |
| Missing | 1 (0.1) |
| Mean age (IQR), y | 27.7 (26-29) |
| Ethnicity |  |
| White | 399 (57.4) |
| Asian | 136 (19.6) |
| Underrepresented in medicine^a^ | 159 (22.9) |
| Missing | 1 (0.1) |
| Specialty^b^ |  |
| Surgical | 133 (19.1) |
| Non-surgical | 562 (80.9) |

^a^Residents were coded as underrepresented in medicine according to the American Association of Medical Colleges definition as "racial and ethnic populations that are underrepresented in the medical profession relative to their numbers in the general population." In this study, this group included interns self-identifying as African American, Arab or Middle Eastern, Latino, Native American, Pacific Islander, other, or multi-racial.

^b^Surgical specialties were assigned based on the American College of Surgeons classification. Specifically, for this study, physicians in the following specialties were classified as “surgical”: General Surgery, Gynecology and Obstetrics, Neurological Surgery, Orthopaedic Surgery, Otolaryngology, Plastic Surgery, Urology, and Other surgical. Physicians from the following specialties were classified as “non-surgical”: Internal Medicine, Pediatrics, Psychiatry, Neurology, Emergency Medicine, Internal Medicine-Pediatrics, Family Medicine, Family Practice, Anesthesiology, Dermatology, Medical Genetics, Nuclear Medicine, Pathology, Physical Medicine & Rehabilitation, Preventative Medicine, Radiation Oncology, Radiology-Diagnostic, Sleep Medicine, and Other Non-surgical.
