# Supplementary material for: Feasibility and acceptability of collecting passive phone usage and sensor data via Apple SensorKit
Source: PLoS One. 2025 Aug 13;20(8):e0329707. doi: 10.1371/journal.pone.0329707 (PMC12349082; doi:10.1371/journal.pone.0329707)
Supplement: S1 File — (DOCX) [file pone.0329707.s002.docx]

Baseline Survey

Thank you for participating! To continue with your study enrollment, please complete the initial survey (15 minutes).

[Only if not known prior to enrollment]

First Name:

Last Name

How many hours have you worked in the PAST WEEK?

How many hours of sleep have you had in the LAST 24 HOURS?

On average, how many hours have you slept per night over the PAST WEEK?

Start date of internship duties:

MOOD SYMPTOMS

For each statement, please mark the response which best represents how often you have been bothered by any of the following problems over the PAST 2 WEEKS

|  | Not at all (0) | Less than half the days (1) | More than half the days (2) | Nearly everyday (3) |
| --- | --- | --- | --- | --- |
| Little interest or pleasure in doing things (1) |  |  |  |  |
| Feeling down, depressed or hopeless (2) |  |  |  |  |
| Trouble falling asleep, staying asleep or sleeping too much (3) |  |  |  |  |
| Feeling tired or having little energy (4) |  |  |  |  |
| Poor appetite or overeating (5) |  |  |  |  |
| Feeling badly about yourself, or that you are a failure, or that you have let yourself or your family down (6) |  |  |  |  |
| Trouble concentrating on things such as reading the newspaper or watching TV (7) |  |  |  |  |
| Moving or speaking so slow that others could have noticed or the opposite, being so fidgety or restless that you have been moving around a lot more than usual (8) |  |  |  |  |
| Thoughts that you would be better off dead or hurting yourself in some way (9) |  |  |  |  |

If you have experienced any of the depressive symptoms described, how difficult have they made it for you to do your work, take care of things at home, or get along with other people?

- Have not experienced any depressive symptoms (4)
- Not difficult at all (0)
- Somewhat difficult (1)
- Very difficult (2)
- Extremely difficult (3)

To the best of your recollection, have any of your first degree relatives (i.e. parents, siblings or children) experienced an episode of depression as described as above?

- Yes (1)
- No (2)

To the best of your recollection, have you EVER experienced an episode of depression (a two week period of your life when you felt down or lost interest or pleasure in your usual activities and also had difficulty concentrating or noticed changes in sleep, appetite, energy or experienced thoughts of death or feelings of guilt)?

- Yes (1)
- No (2)

How old were you when you first experienced an episode of depression?

Please indicate when the episode(s) of depression took place (check all that apply).

- High school or before (1)
- Between high school and college (2)
- During college (3)
- Between college and medical school (4)
- During medical school (5)

Have you EVER received medication or psychotherapy for the treatment of depression?

- Yes (1)
- No (2)

Which treatment(s) did you receive (check all that apply)?

- Medication (1)
- Psychotherapy (2)
- Other (3) ____________________

Are you CURRENTLY taking any of the following medications (check all that apply)?

- None (1)
- Prescription analgesics (2)
- Sedatives or Hypnotics (3)
- Antidepressants (4)
- Mood stabilizers (5)
- Antipsychotics (6)
- Stimulants (7)
- Other (8) ____________________

In the past three months, how often have you used the following substances?

|  | Never | Once or Twice | Monthly | Weekly | Daily or Almost Daily |
| --- | --- | --- | --- | --- | --- |
| Tobacco products (cigarettes, chewing tobacco, cigars, etc.) (1) |  |  |  |  |  |
| Alcoholic beverages (beer, wine, spirits, etc.) (2) |  |  |  |  |  |
| Cannabis (marijuana, pot, grass, hash, etc.) (3) |  |  |  |  |  |

Are you CURRENTLY participating in psychotherapy?

- Yes (1)
- No (2)

ANXIETY SYMPTOMS

Over the LAST TWO WEEKS, how often have you been bothered by the following problems?

|  | Not at all (0) | Less than half the days (1) | More than half the days (2) | Nearly everyday (3) |
| --- | --- | --- | --- | --- |
| Feeling anxious, nervous, or on edge (1) |  |  |  |  |
| Not being able to stop or control worrying (2) |  |  |  |  |
| Worrying too much about different things (3) |  |  |  |  |
| Trouble relaxing (4) |  |  |  |  |
| Being so restless that it's hard to sit still (5) |  |  |  |  |
| Becoming easily annoyed or irritable (6) |  |  |  |  |
| Feeling afraid as if something awful might happen (7) |  |  |  |  |

Please indicate if you have experienced any of the following events during the PAST 3 MONTHS (select all that apply).

- Death of a family member, significant other or close friend (1)
- You developed a disabling illness or injury lasting a month or more (2)
- A disabling physical illness or injury started or got worse in a family member, significant other or close friend (3)
- A relationship with an intimate cohabiting partner ended (4)
- You were involved in a physically violent relationship (5)
- You suffered a significant financial loss or loss of property (6)
- You had problems with debt i.e. having items repossessed, not having enough money to pay household expenses, lacking money for medical expenses or difficulty paying bills (7)
- You were physically assaulted or attacked (8)
- You got married (9)
- You learned that you or your partner were pregnant (11)
- You had a child (10)
- None of the above

INTERPERSONAL STYLE

For each statement, please mark the response which best represents your level of agreement with the statement. Please choose the response that CURRENTLY best describes you.

|  | Strongly agree (4) | Agree (3) | Neutral (2) | Disagree (1) | Strongly disagree (0) |
| --- | --- | --- | --- | --- | --- |
| I rarely feel anxious or nervous (1) |  |  |  |  |  |
| I rarely experience strong emotions (2) |  |  |  |  |  |
| I am not a worrier (3) |  |  |  |  |  |
| I often worry about things that might go wrong (4) |  |  |  |  |  |
| Frightening thoughts sometimes come into my head (5) |  |  |  |  |  |
| I rarely feel lonely or blue (6) |  |  |  |  |  |
| Too often, when things go wrong, I get discouraged and feel like giving up (7) |  |  |  |  |  |
| I am seldom sad or depressed (8) |  |  |  |  |  |
| I often feel helpless and want someone else to solve my problems (9) |  |  |  |  |  |
| When I am having my favorite foods, I tend to eat too much (10) |  |  |  |  |  |
| At times I have been so ashamed that I just wanted to hide (11) |  |  |  |  |  |
| When I'm under a great deal of stress, sometimes I feel like I'm going to pieces (12) |  |  |  |  |  |
| I often feel inferior to others (13) |  |  |  |  |  |
| I feel comfortable in the presence of my bosses or other authorities (14) |  |  |  |  |  |

EARLY FAMILY ENVIRONMENT

These are questions about your childhood and early adolescence (age 5 - 15). Please think about your family life while answering the questions in this section.

|  | 1 Not at all (1) | 2 (2) | 3 (3) | 4 (4) | 5 (5) | 6 Very often (6) |
| --- | --- | --- | --- | --- | --- | --- |
| How often did a parent or other adult in the household make you feel that you were loved, supported and cared for? (1) |  |  |  |  |  |  |
| How often did a parent or other adult in the household swear at you, insult you, put you down or act in a way that made you feel threatened? (2) |  |  |  |  |  |  |
| How often did a parent or other adult in the household express physical affection for you, such as hugging or other physical gestures of warmth and affection? (3) |  |  |  |  |  |  |
| How often did a parent or other adult in the household push, slap or shove you? (4) |  |  |  |  |  |  |
| Would you say that the household you grew up in was well-organized and well-managed? (5) |  |  |  |  |  |  |
| In your childhood, did you live with anyone who was a problem drinker or alcoholic or who used illicit drugs? (6) |  |  |  |  |  |  |
| How often would you say that a parent or other adult in the household behaved violently toward a family member or visitor in your home? (7) |  |  |  |  |  |  |
| How often would you say that there was quarreling, arguing or shouting between your parents? (8) |  |  |  |  |  |  |
| How often would you say there was quarreling, arguing, or shouting between a parent and you? (9) |  |  |  |  |  |  |
| How often would you say there was quarreling, arguing, or shouting between a parent and one of your siblings? (10) |  |  |  |  |  |  |
| How often would you say there was quarreling, arguing, or shouting between your sibling(s) and you? (11) |  |  |  |  |  |  |
| Would you say the household you grew up in was chaotic and disorganized? (12) |  |  |  |  |  |  |
| How often would you say you were neglected while you were growing up, that is, left on your own to fend for yourself? (13) |  |  |  |  |  |  |

SOCIAL SUPPORT Below is a list of ways that you think about the support that you are getting from your family, friends, and significant others. Please indicate the extent to which you agree with each item by circling the appropriate number.

|  | 1 Strongly disagree (1) | 2 (2) | 3 (3) | 4 (4) | 5 Strongly agree (5) |
| --- | --- | --- | --- | --- | --- |
| There is a special person who is around when I am in need (1) |  |  |  |  |  |
| There is a special person with whom I can share my joys and sorrows (2) |  |  |  |  |  |
| My family really tries to help me (3) |  |  |  |  |  |
| I get the emotional help and support I need from my family (4) |  |  |  |  |  |
| I have a special person who is a real source of comfort for me (5) |  |  |  |  |  |
| My friends really try to help me (6) |  |  |  |  |  |
| I can count on my friends when things go wrong (7) |  |  |  |  |  |
| I can talk about my problems with my family (8) |  |  |  |  |  |
| I have friends with whom I can share my joys and sorrows (9) |  |  |  |  |  |
| There is a special person in my life who cares about my feelings (10) |  |  |  |  |  |
| My family is willing to help me make decisions (11) |  |  |  |  |  |
| I can talk about my problems with my friends (12) |  |  |  |  |  |

Where do/did you go for medical school?

If you are still in medical school, what is your current clerkship?

- Sub-Internship (1)
- Elective (2)
- Vacation (3)

Internship Program

Start typing to search for your internship specialty. Once selected, you will be prompted to search for your state, city and institution.

DEMOGRAPHICS

Date of Birth (MM/DD/YYYY)

Sex assigned at birth:

- Male (1)
- Female (2)

Gender identity:

- Man (1)
- Woman (2)
- Transgender (3)
- Non-binary (4)
- Other_____ (5)
- Prefer not to say

Which of the following best describes your sexual orientation?

- Heterosexual (1)
- Gay / Lesbian (2)
- Bisexual (3)
- Queer (6)
- Other (4) ____________________
- Prefer not to say (5)

Current marital status

- Not in a committed relationship
- In a committed relationship
- Engaged (2)
- Married (3)
- Separated / Divorced / Widowed (6)

*[BRANCH: If “In a committed relationship,” “Engaged,” or “Married”]*

Please indicate your partner’s expected employment status as of July 1, 2023:

- Student
- Physician
- Other medical professional (e.g. dentist, nurse, pharmacist)
- Non-medical professional (e.g. business person, engineer)
- Other occupation_____
- Not a student or working for pay outside the home

*[BRANCH: If “student”]*

Your partner’s degree program:

- Medical degree (e.g. MD, DO)
- Other professional degree (e.g. JD, MBA)
- Advanced academic degree (e.g. MA, PhD)
- Other______

*[BRANCH: If “physician”]*

Please indicate your partner’s position as of July 1, 2023:

- Intern
- Other house officer
- Attending

Will you be living with a significant other during your INTERN YEAR?

- Yes (1)
- No (2)

How many children do you have?

- 0
- 1
- 2
- 3 or more

Ethnicity (check all that apply)

- White (1)
- Black / African American (2)
- Latino / Hispanic (3)
- Asian (e.g. Indian, Chinese) (4)
- Arab / Middle Eastern (9)
- Native American (5)
- Pacific Islander (6)
- Other (7) ____________________

Asian ethnicity (check all that apply)

- Indian (1)
- Chinese (2)
- Korean (3)
- Vietnamese (4)
- Japanese (5)
- Pakistani (6)
- Filipino (7)
- Thai (8)
- Cambodian (9)
- Indonesian (10)
- Other (11) ____________________

Country or region of birth

[BRANCH: If a country/region other than “United States”]

At what age did you first move to the United States?

What personal or professional factors do you expect to most influence your well-being during intern year? You may also share any additional information relevant to the study. (Optional)

As part of this study, we ask that you wear a compatible fitness tracker* to collect sleep, steps, and other activity data prior to and during internship. Please choose the appropriate response below:

- I have an Apple Watch I can use for the study
- I have a Fitbit I can use for the study
- I have a Garmin I can use for the study
- I do not have a compatible fitness tracker* I can use for the study

[BRANCH: If “I do not have a compatible fitness tracker…”]

*Any Apple Watch, Fitbit, or Garmin fitness tracker or smartwatch

Upon completing enrollment, we will mail you a fitness tracker to use for the study. Please indicate your preferred device below (as long as supplies last).

- Fitbit Inspire 2 (sleek profile, 10-day battery life)
- Fitbit Charge 4 (more features)
- Apple Watch [insert model] (if available)

Please provide the best email address to reach you now and after your internship starts.

Please provide your CURRENT mailing address below so that we may send you a Fitbit (if applicable) and home DNA collection kit within the next 2-4 weeks. Your name and address will not be connected to your survey responses. Upon receiving your DNA kit, you will be instructed to return the DNA sample without any identifying information so that your name and address will not be connected to your genetic information.

Name:

Street Address 1:

Street Address 2:

City:

State:

Zip Code:

Until what date will your CURRENT address be valid (MM/DD/YYYY)?

If you will be moving within the next couple of months and already know your NEW mailing address, please provide it below. If you do not yet know your exact address, you will have the opportunity to provide us with an update when you complete the first follow-up survey in the fall. You may also send us an email with your updated contact information at any time.

Street Address 1:

Street Address 2:

City:

State:

Zip Code:

Thank you for participating in the Intern Health Study! If you have any questions, please feel free to contact us at any time at Intern_Health@med.umich.edu.

If you are finished with the survey, please click “Submit” and follow the instructions on the next page to complete study enrollment.
