# Supplementary material for: Feasibility and acceptability of collecting passive phone usage and sensor data via Apple SensorKit
Source: PLoS One. 2025 Aug 13;20(8):e0329707. doi: 10.1371/journal.pone.0329707 (PMC12349082; doi:10.1371/journal.pone.0329707)
Supplement: S2 File — (DOCX) [file pone.0329707.s003.pdf]

11:35

Cancel

Enable Research Sensor & Usage Data

Your devices are not currently collecting any research data.

In order to share research data with "MyDataHelps", you need to turn on Sensor & Usage Data collection in Settings.

Open Settings

11:38

Cancel

About Sensor & Usage Data

This sensitive data can help apps and studies further their research. Your device can collect this data on behalf of apps and studies you authorize while still protecting your privacy.

Next

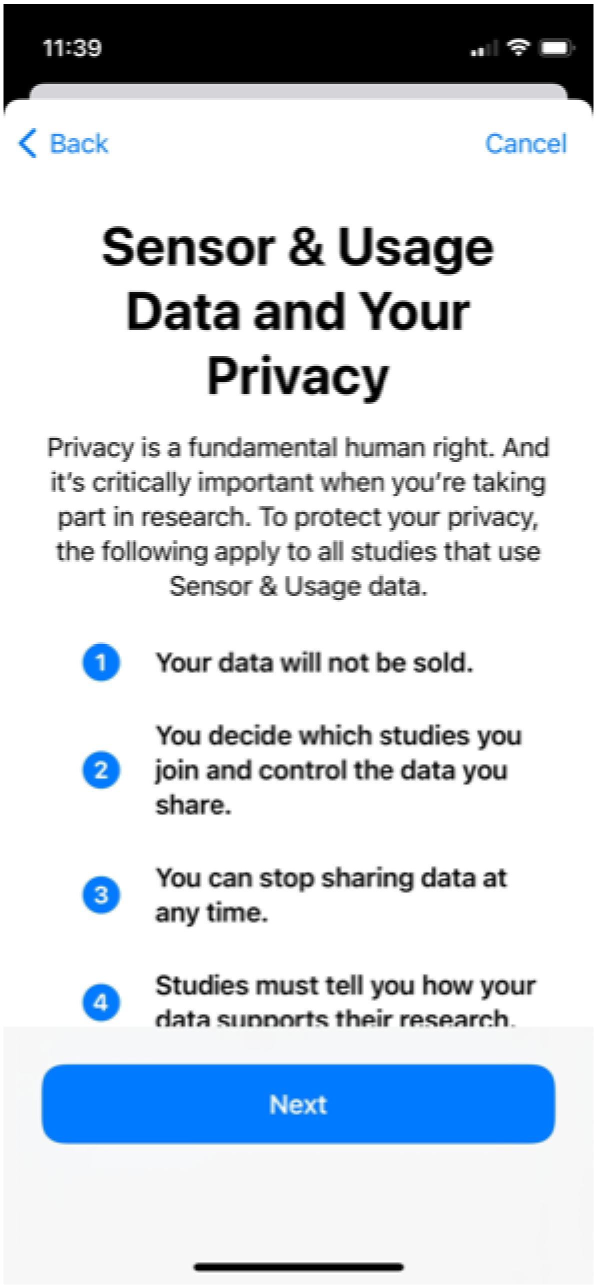
11:39
Back
Cancel
Sensor & Usage Data and Your Privacy
Privacy is a fundamental human right. And it's critically important when you're taking part in research. To protect your privacy, the following apply to all studies that use Sensor & Usage data.
1 Your data will not be sold.
2 You decide which studies you join and control the data you share.
3 You can stop sharing data at any time.
4 Studies must tell you how your
Next

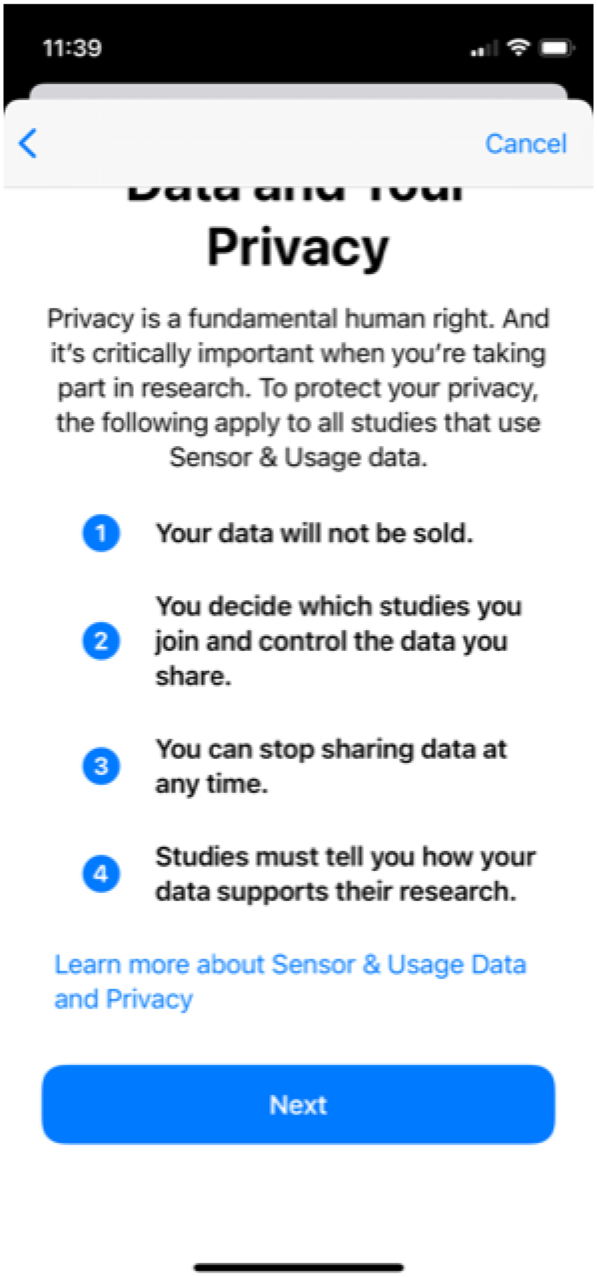
11:39
Cancel
Privacy
Privacy is a fundamental human right. And it's critically important when you're taking part in research. To protect your privacy, the following apply to all studies that use Sensor & Usage data.
1 Your data will not be sold.
2 You decide which studies you join and control the data you share.
3 You can stop sharing data at any time.
4 Studies must tell you how your data supports their research.
Learn more about Sensor & Usage Data and Privacy
Next

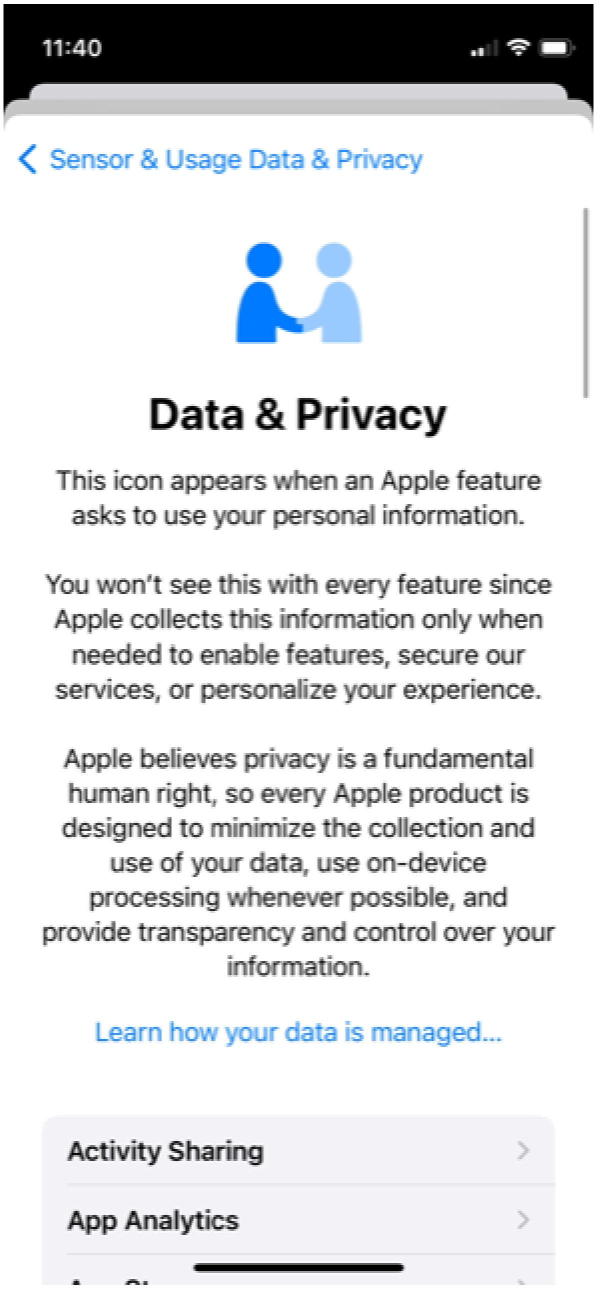
11:40
Sensor & Usage Data & Privacy
Data & Privacy
This icon appears when an Apple feature asks to use your personal information.
You won't see this with every feature since Apple collects this information only when needed to enable features, secure our services, or personalize your experience.
Apple believes privacy is a fundamental human right, so every Apple product is designed to minimize the collection and use of your data, use on-device processing whenever possible, and provide transparency and control over your information.
Learn how your data is managed...
Activity Sharing
App Analytics

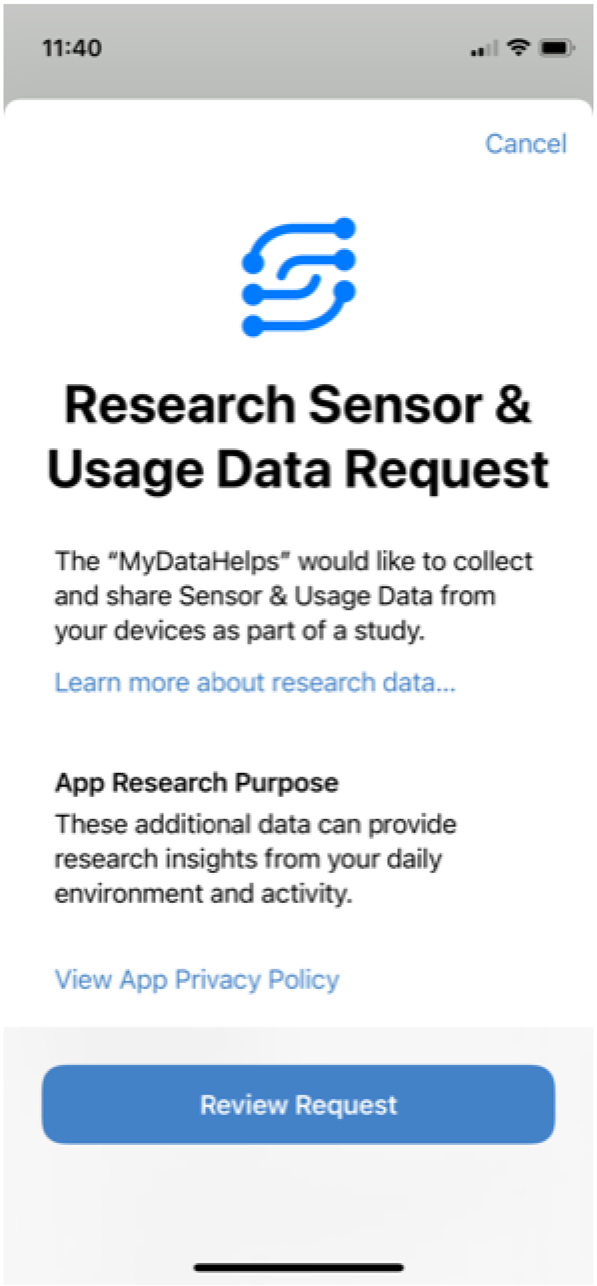
11:40
Cancel
Research Sensor & Usage Data Request
The "MyDataHelps" would like to collect and share Sensor & Usage Data from your devices as part of a study.
Learn more about research data...
App Research Purpose
These additional data can provide research insights from your daily environment and activity.
View App Privacy Policy
Review Request

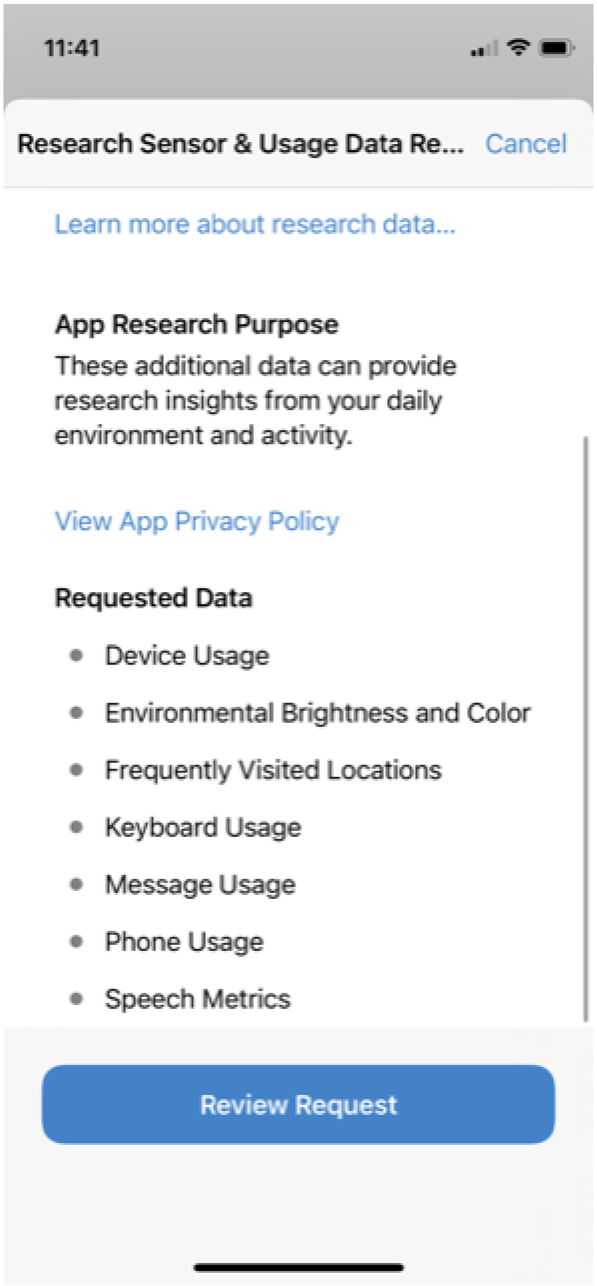
11:41
Research Sensor & Usage Data Re...
Cancel
Learn more about research data...
App Research Purpose
These additional data can provide research insights from your daily environment and activity.
View App Privacy Policy
Requested Data
Device Usage
Environmental Brightness and Color
Frequently Visited Locations
Keyboard Usage
Message Usage
Phone Usage
Speech Metrics
Review Request

11:41

< Back 1 of 7 Cancel

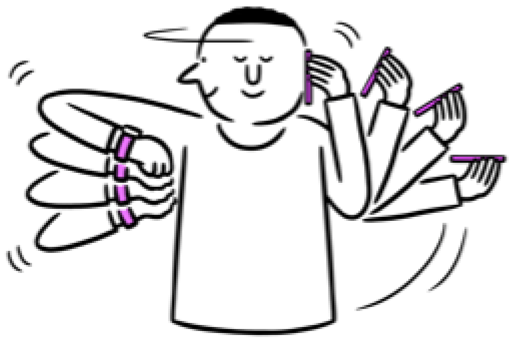

IPHONE & APPLE WATCH

Device Usage

How the study will use this data:

To learn how general information about device usage may relate to health.

What is collected:

- The types of apps you use, the number of unique apps you use, how long you use them, and how often you return to the same app.
- The number of notifications you receive, the type of app that sent them, and if you interact with them.

11:42

< Back 1 of 7 Cancel

- The number of notifications you receive, the type of app that sent them, and if you interact with them.
- The type of websites you visit such as Travel, News, or Weather and how long you're on a site.
- The number of times you unlock your iPhone or pick up your iPhone and the approximate time of day.
- The number of times you raise your wrist if wearing Apple Watch and the approximate time of day.
- Whether or not text has been input into an app along with the type of input such as a keyboard, Apple Pencil, or dictation.
- The amount of time a device is charging.
- How often and for how long you're actively using a device.
- The type of motion or activity you're doing such as walking or biking.

What is not collected:

- The specific names of apps you download and use from the App Store.

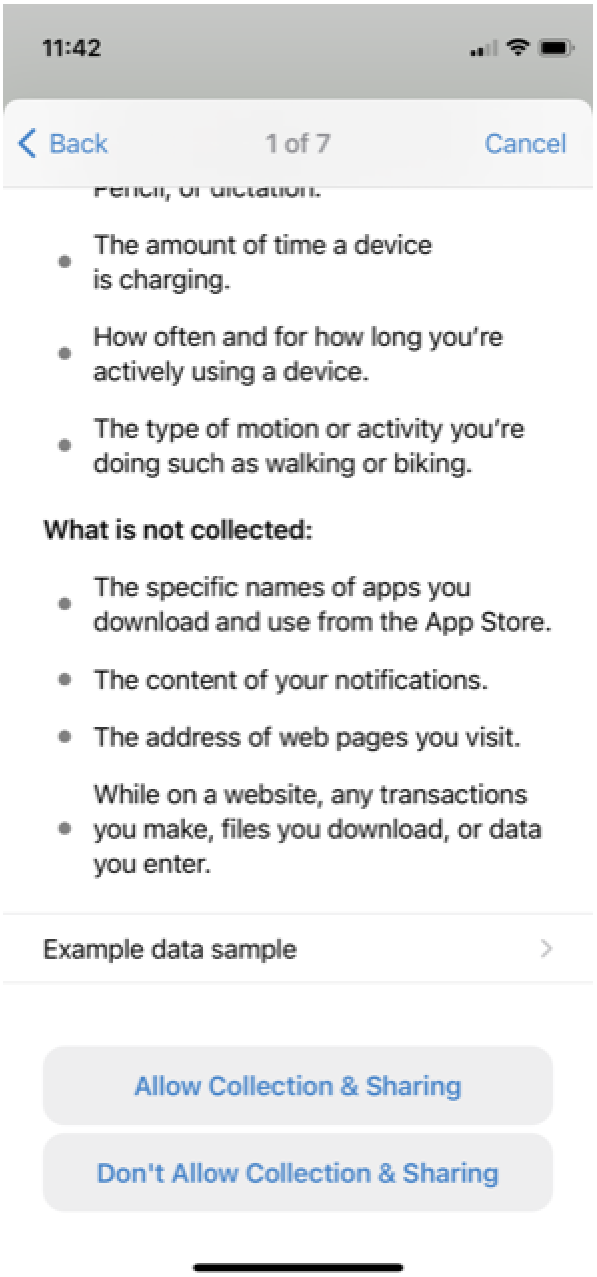
11:42
Back
1 of 7
Cancel
The amount of time a device is charging.
How often and for how long you're actively using a device.
The type of motion or activity you're doing such as walking or biking.
What is not collected:
The specific names of apps you download and use from the App Store.
The content of your notifications.
The address of web pages you visit.
While on a website, any transactions you make, files you download, or data you enter.
Example data sample
Allow Collection & Sharing
Don't Allow Collection & Sharing

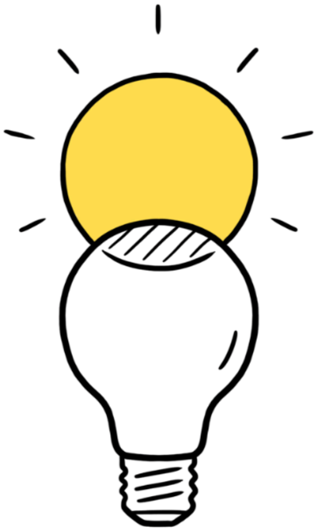

IPHONE & APPLE WATCH

Environmental Brightness and Color

How the study will use this data:

To learn how setting (indoor and outdoor) and other environmental factors may impact health.

What is collected:

- Brightness or color measurements from the light sensors in your devices. Data like this can be used to identify

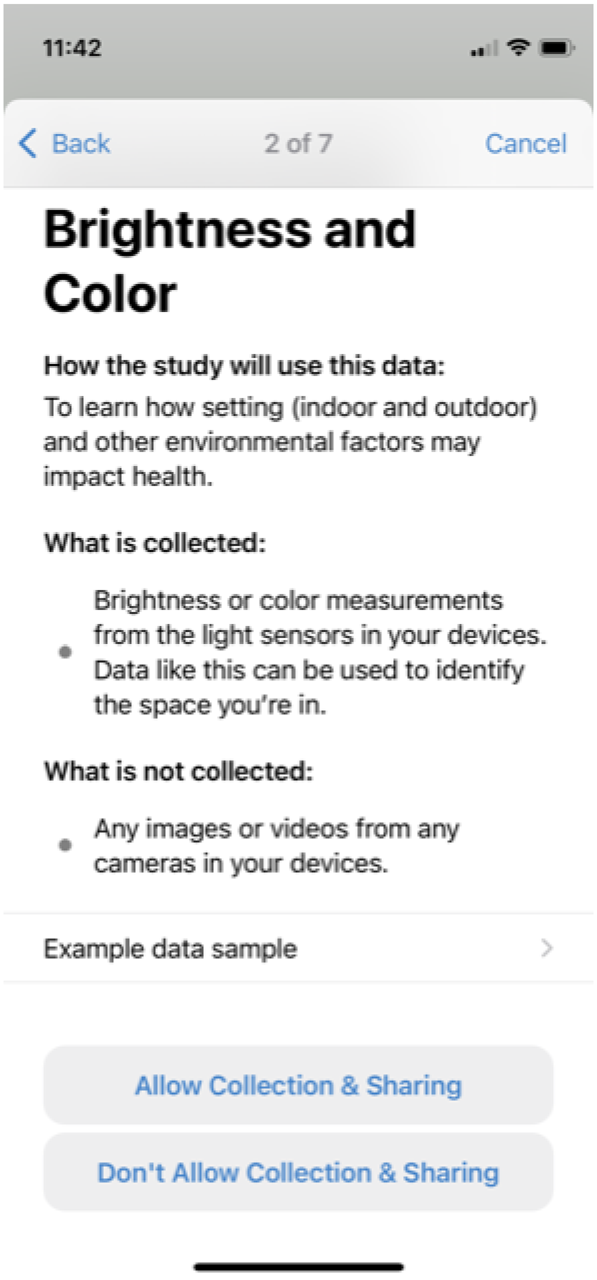
11:42
Back
2 of 7
Cancel
Brightness and Color
How the study will use this data:
To learn how setting (indoor and outdoor) and other environmental factors may impact health.
What is collected:
Brightness or color measurements from the light sensors in your devices. Data like this can be used to identify the space you're in.
What is not collected:
Any images or videos from any cameras in your devices.
Example data sample
Allow Collection & Sharing
Don't Allow Collection & Sharing

11:43

< Back Cancel

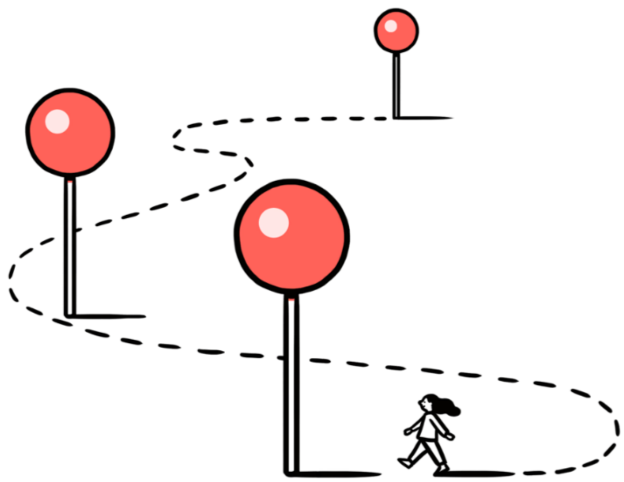

IPHONE ONLY

Frequently Visited Locations

How the study will use this data:

To learn about the impact that certain locations and environments may have on health.

What is collected:

- Frequently visited locations but only after they are given an anonymized identifier.
- The distance of an anonymized [illegible]

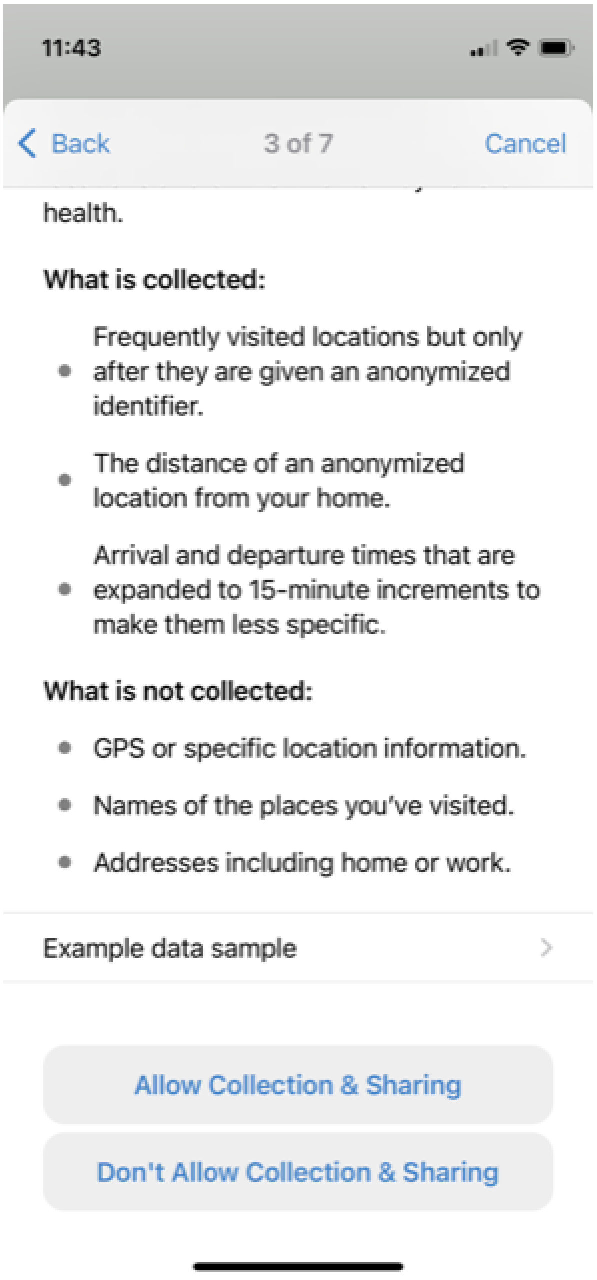
11:43
Back
3 of 7
Cancel
health.
What is collected:
Frequently visited locations but only after they are given an anonymized identifier.
The distance of an anonymized location from your home.
Arrival and departure times that are expanded to 15-minute increments to make them less specific.
What is not collected:
GPS or specific location information.
Names of the places you've visited.
Addresses including home or work.
Example data sample
Allow Collection & Sharing
Don't Allow Collection & Sharing

11:43

< Back 4 of 7 Cancel

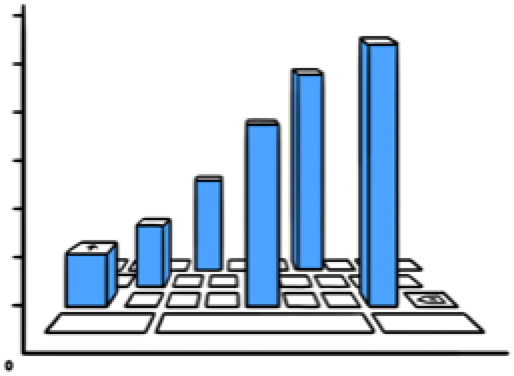

IPHONE ONLY

Keyboard Usage

How the study will use this data:

To learn how keyboard usage and trends may relate to health.

What is collected:

- The length of words you type or swipe, your speed, your accuracy, and the kind of errors you make.
- Data about the keyboard, its orientation, and your screen size.
- The number of words and emoji you type that convey emotion

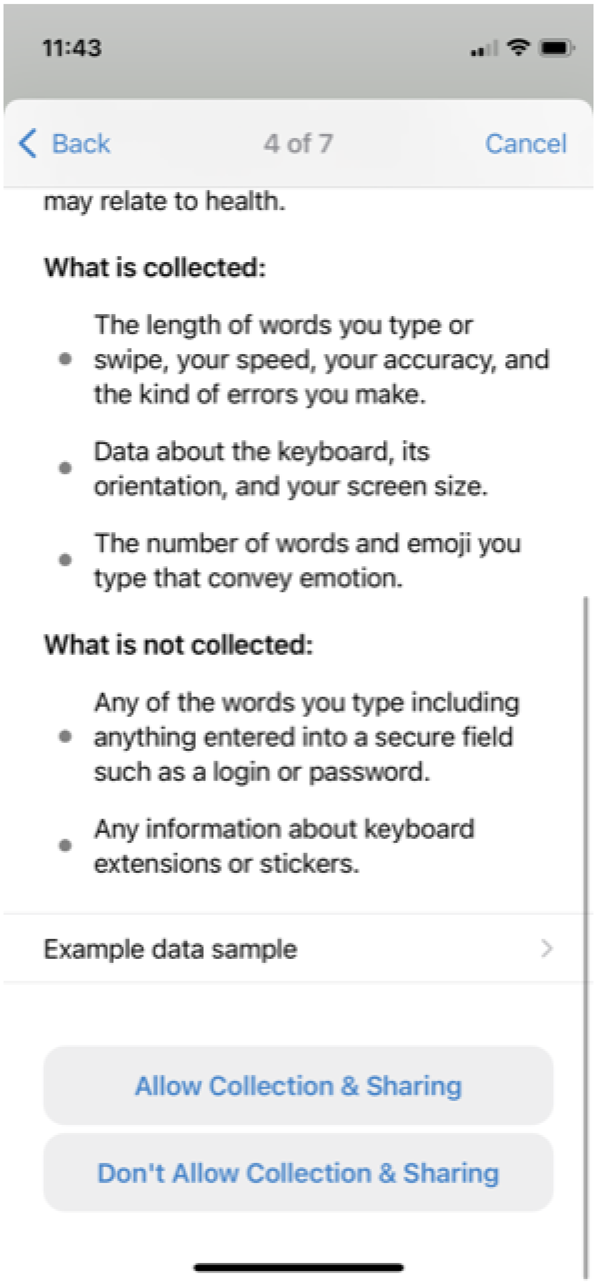
11:43
Back
4 of 7
Cancel
may relate to health.
What is collected:
The length of words you type or swipe, your speed, your accuracy, and the kind of errors you make.
Data about the keyboard, its orientation, and your screen size.
The number of words and emoji you type that convey emotion.
What is not collected:
Any of the words you type including anything entered into a secure field such as a login or password.
Any information about keyboard extensions or stickers.
Example data sample
Allow Collection & Sharing
Don't Allow Collection & Sharing

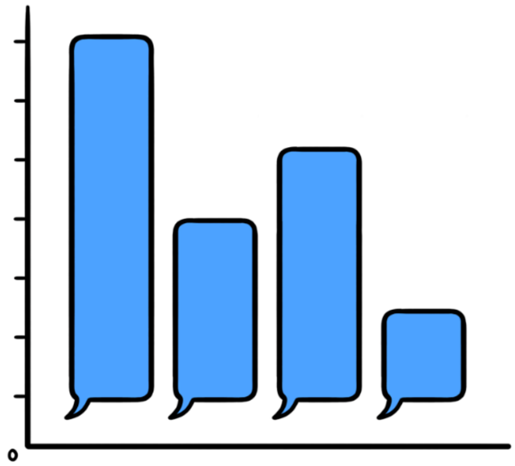

IPHONE ONLY

Message Usage

How the study will use this data:

To learn how messaging usage may relate to health.

What is collected:

- The total number of messages during different parts of the day.
- The number of individuals you message.

What is not collected:

The names or phone numbers of

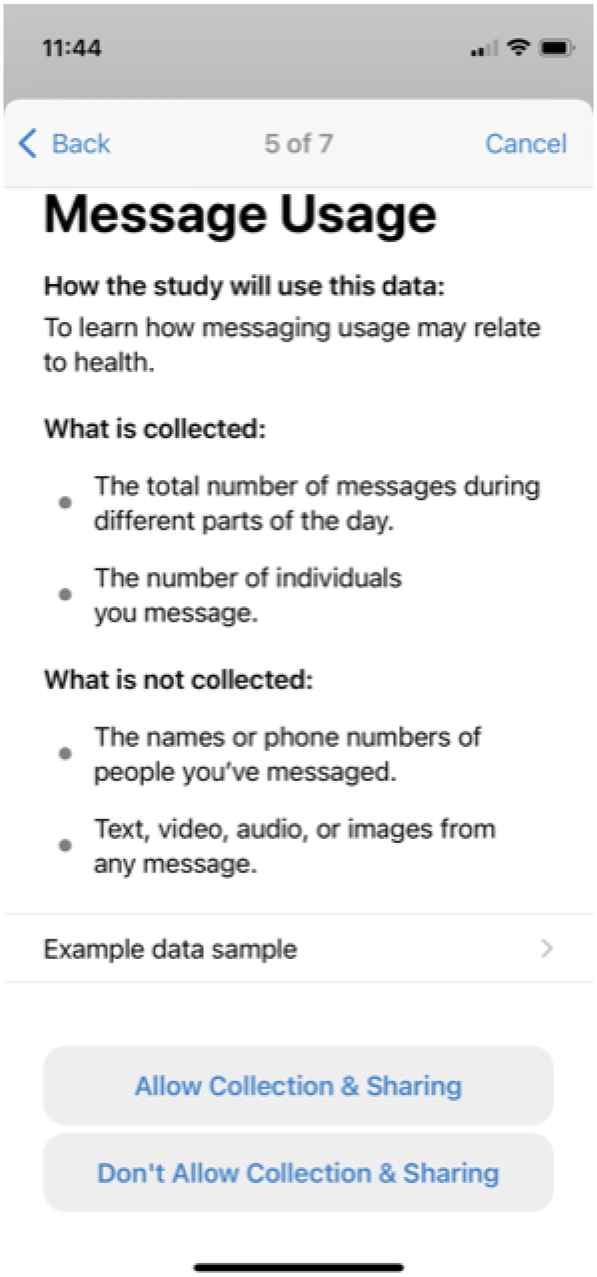
11:44
Back
5 of 7
Cancel
Message Usage
How the study will use this data:
To learn how messaging usage may relate to health.
What is collected:
The total number of messages during different parts of the day.
The number of individuals you message.
What is not collected:
The names or phone numbers of people you've messaged.
Text, video, audio, or images from any message.
Example data sample
Allow Collection & Sharing
Don't Allow Collection & Sharing

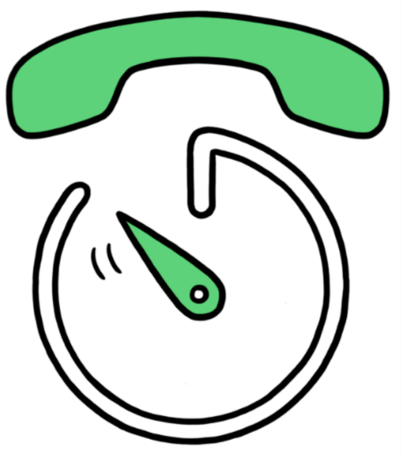

IPHONE ONLY

Phone Usage

How the study will use this data:

To learn how general information about phone usage may relate to health.

What is collected:

- The number of incoming and outgoing calls each day.
- The number of people you've contacted but none of their personal information such as a name or phone number.

[illegible]

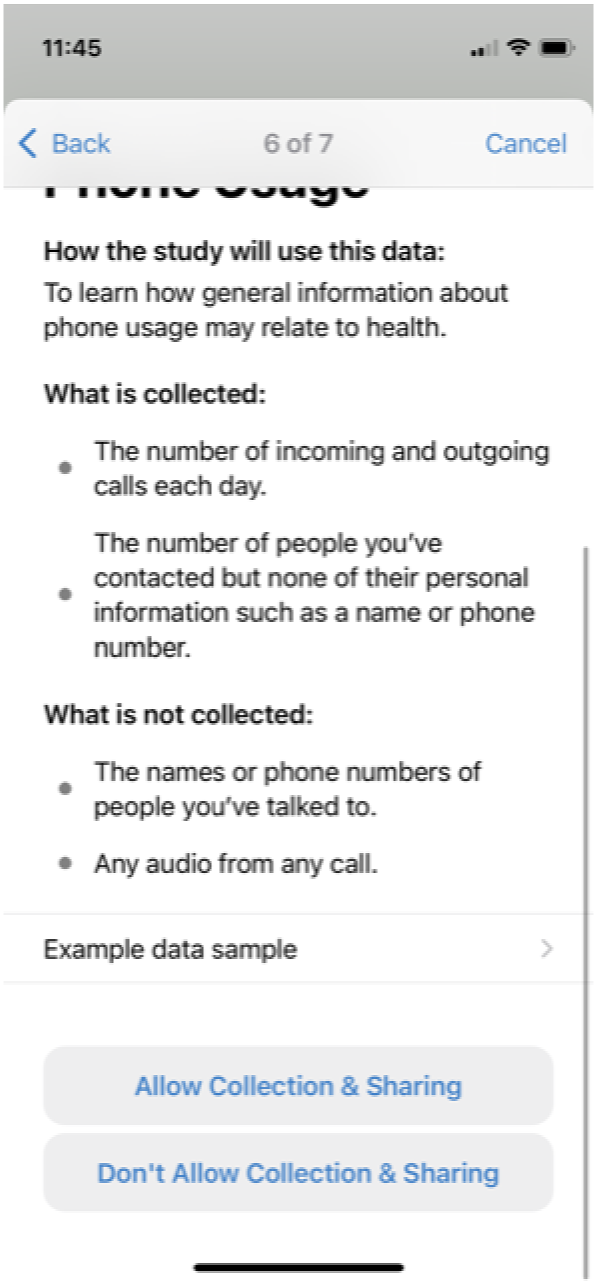
11:45
Back
6 of 7
Cancel
How the study will use this data:
To learn how general information about phone usage may relate to health.
What is collected:
The number of incoming and outgoing calls each day.
The number of people you've contacted but none of their personal information such as a name or phone number.
What is not collected:
The names or phone numbers of people you've talked to.
Any audio from any call.
Example data sample
Allow Collection & Sharing
Don't Allow Collection & Sharing

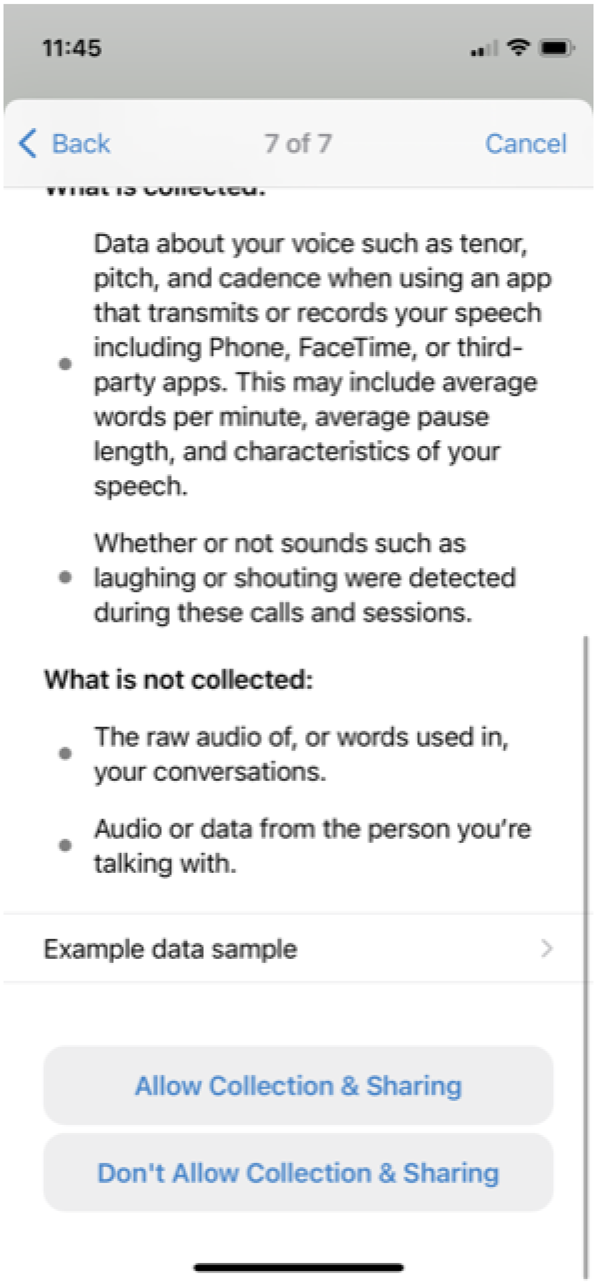
11:45
Back
7 of 7
Cancel
Data about your voice such as tenor, pitch, and cadence when using an app that transmits or records your speech including Phone, FaceTime, or third-party apps. This may include average words per minute, average pause length, and characteristics of your speech.
Whether or not sounds such as laughing or shouting were detected during these calls and sessions.
What is not collected:
The raw audio of, or words used in, your conversations.
Audio or data from the person you're talking with.
Example data sample
Allow Collection & Sharing
Don't Allow Collection & Sharing

11:46

Back Cancel

Authorization Review

You've given "MyDataHelps" access to:

- Device Usage
- Environmental Brightness and Color
- Frequently Visited Locations
- Keyboard Usage
- Message Usage
- Phone Usage
- Speech Metrics

Data Collection and Access

You can turn off access to this data at any time in Privacy settings or by deleting MyDataHelps.

Done
